# Chloroquine ameliorates carbon tetrachloride-induced acute liver injury in mice via the concomitant inhibition of inflammation and induction of apoptosis

**DOI:** 10.1038/s41419-018-1136-2

**Published:** 2018-11-26

**Authors:** Chongshan Dai, Xilong Xiao, Daowen Li, Sun Tun, Ying Wang, Tony Velkov, Shusheng Tang

**Affiliations:** 1 College of Veterinary Medicine, China Agricultural University, No. 2 Yuanmingyuan West Road, Beijing, 100193 P. R. China; 2 Department of Pharmacology & Therapeutics, School of Biomedical Sciences, Faculty of Medicine, Dentistry and Health Sciences, The University of Melbourne, Parkville, Victoria 3010 Australia; 3 Drug Delivery, Disposition and Dynamics, Monash Institute of Pharmaceutical Sciences, Monash University, 381 Royal Parade, Parkville, Victoria 3052 Australia

## Abstract

This is the first study to investigate the hepatoprotective effect of CQ on acute liver injury caused by carbon tetrachloride (CCl_4_) in a murine model and the underlying molecular mechanisms. Ninety-six mice were randomly divided into the control (*n* = 8), CQ (*n* = 8), CCl_4_ (*n* = 40), and CCl_4_ + CQ (*n* = 40) treatment groups. In the CCl_4_ group, mice were intraperitoneally (i.p) injected with 0.3% CCl_4_ (10 mL/kg, dissolved in olive oil); in the CCl_4_ + CQ group, mice were i.p injected with CQ at 50 mg/kg at 2, 24, and 48 h before CCl_4_ administration. The mice in the control and CQ groups were administered with an equal vehicle or CQ (50 mg/kg). Mice were killed at 2, 6, 12, 24, 48 h post CCl_4_ treatment and their livers were harvested for analysis. The results showed that CQ pre-treatment markedly inhibited CCl_4_-induced acute liver injury, which was evidenced by decreased serum transaminase, aspartate transaminase and lower histological scores of liver injury. CQ pretreatment downregulated the CCl_4_-induced hepatic tissue expression of high-mobility group box 1 (HMGB1) and the levels of serum HMGB1 as well as IL-6 and TNF-α. Furthermore, CQ pre-treatment inhibited autophagy, downregulated NF-kB expression, upregulated p53 expression, increased the ratio of Bax/Bcl-2, and increased the activation of caspase-3 in hepatic tissue. This is the first study to demonstrate that CQ ameliorates CCl_4_-induced acute liver injury via the inhibition of HMGB1-mediated inflammatory responses and the stimulation of pro-apoptotic pathways to modulate the apoptotic and inflammatory responses associated with progress of liver damage.

## Introduction

Liver disease is a global health problem, in particular, acute liver injury is associated with high mortality rates^1,2^. The molecular processes underlying the pathogenesis of acute liver injury are known to involve a complex interplay of oxidative stress, apoptosis, autophagy, and necrosis^3,4^. Nuclear factor high-mobility group box 1 (HMGB1) appears to regulate oxidative stress, inflammatory signaling, and autophagy in hepatocytes^5,6^. Not surprisingly, HMGB1 plays a critical role in a wide array of liver diseases, such as liver ischemia‐reperfusion injury, alcoholic liver disease, cholestasis, and drug-induced liver injury^7–10^. The activation of HMGB1 is purported to regulate the NF-κB and mitogen-activated protein kinase (MAPK) pathways via the modulation of toll-like receptors (TLR) −2, 4, and 9, which in turn cascade to regulate the expression of inflammatory mediators, such as tumor necrosis factor (TNF)-α and interleukin (IL)-1β, IL-6, and cyclooxygenase-2^11^.

Carbon tetrachloride (CCl_4_)-induced acute liver injury in murine models is widely used to investigate potential therapeutic strategies due to its similarities with acute chemical liver injury in humans^3,12–15^. CCl_4_ is metabolized by the cytochrome P450 enzymes to form reactive intermediates, such as trichloromethyl-free radicals and peroxyl radical, which then initiate lipid peroxidation and cellular damage. Recently, Chen et al. demonstrated that hepatic HMGB1 expression played a critical role in CCl_4_-induced acute liver injury, and the blockade of HMGB1 (via a HMGB1-neutralizing antibody) could reduce oxidative stress and inflammation^14^. Similarly, Li et al. showed quercetin could reduce CCl_4_-induced liver fibrosis by inhibiting the expression of HMGB1^8^.

Chloroquine (CQ) is an old antimalarial drug that has been repurposed as an antiinflammatory to treat rheumatoid arthritis, systemic lupus erythematosus, and Sjögren’s syndrome^16^. There is a growing body of evidence suggesting that CQ promotes apoptosis via the inhibition of autophagy^17–20^. A study from Yang et al. showed that CQ pretreatment could improve lipopolysaccharide (LPS)-induced mortality in mice by inhibiting the HMGB1 and NF-κB-mediated inflammatory pathways^18^. CQ has been shown to play dual roles in the process of liver ischemia reperfusion injury due to its antiinflammatory and pro-apoptotic activities^19^.

In the present study, we investigated the impact of CQ pretreatment on CCl_4_-induced acute liver injury in a murine model. Our findings revealed CQ pretreatment improved CCl_4_-induced lethal liver failure death. The hepatoprotective effect of CQ involves the concomitant inhibition of HMGB1 and NF-κB-mediated inflammatory pathways, inhibition of autophagy, and the activation of pro-apoptotic pathways. These findings highlight the clinical potential of CQ as a treatment strategy for acute liver injury in patients.

## Results

### Chloroquine pre-treatment attenuates CCl_4_-induced acute liver injury and mortality in mice

**Fig. 1:**
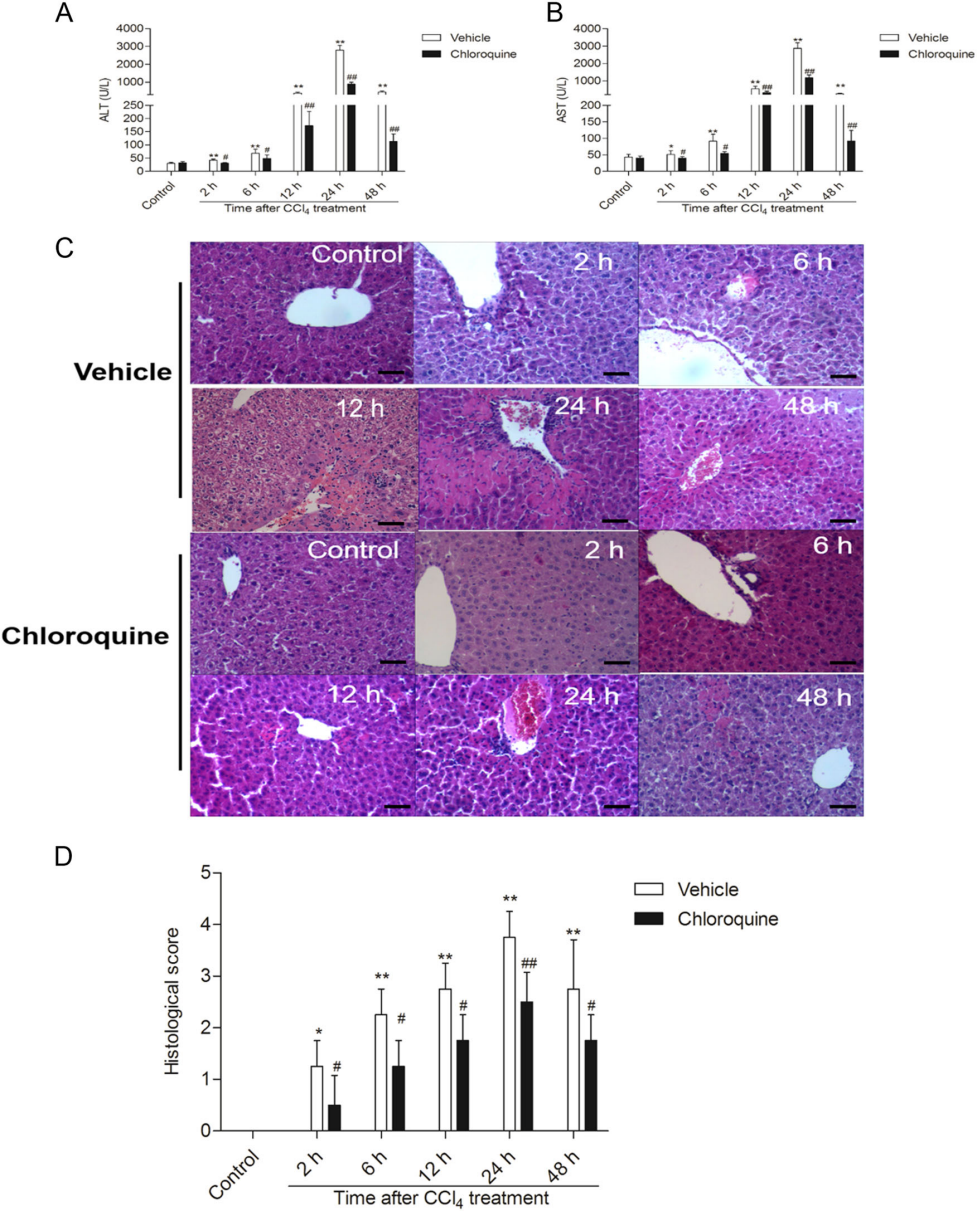
Chloroquine pretreatment attenuates CCl_4_-induced acute liver injury in mice. **a** Time course of ALT serum levels (*n* = 8). **b** Time course of AST serum levels (*n* = 8). **c** Representative histopathological images of H&E-stained liver sections from CCl_4_-treated mice pretreated with vehicle or CQ. Bar = 100 μm. **d** The histological scores for liver sections from CCl_4_-treated mice pretreated with vehicle or CQ (*n* = 4). Data are presented as mean ± SD. *P < 0.05 and **P < 0.01, compared with the control group; ^#^*P* < 0.05 and ^##^*P* < 0.01, compared with the CCl_4_ + vehicle group

First, we assessed the time course of the hepatoprotective effect of CQ against CCl_4_-induced acute liver injury using the levels of serum ALT and AST, and liver histology as endpoints. Figure 1a, b shows the time course of the CCl_4_-induced changes of serum ALT and AST levels. Compared with the CCl_4_ + vehicle group, the mice from the CCl4 + CQ group displayed significantly attenuated serum ALT and AST levels (all *P* *<* 0.05 or 0.01). Mice from the CQ-only treatment group displayed comparable serum ALT and AST levels with the vehicle-treated control group.

**Fig. 2:**
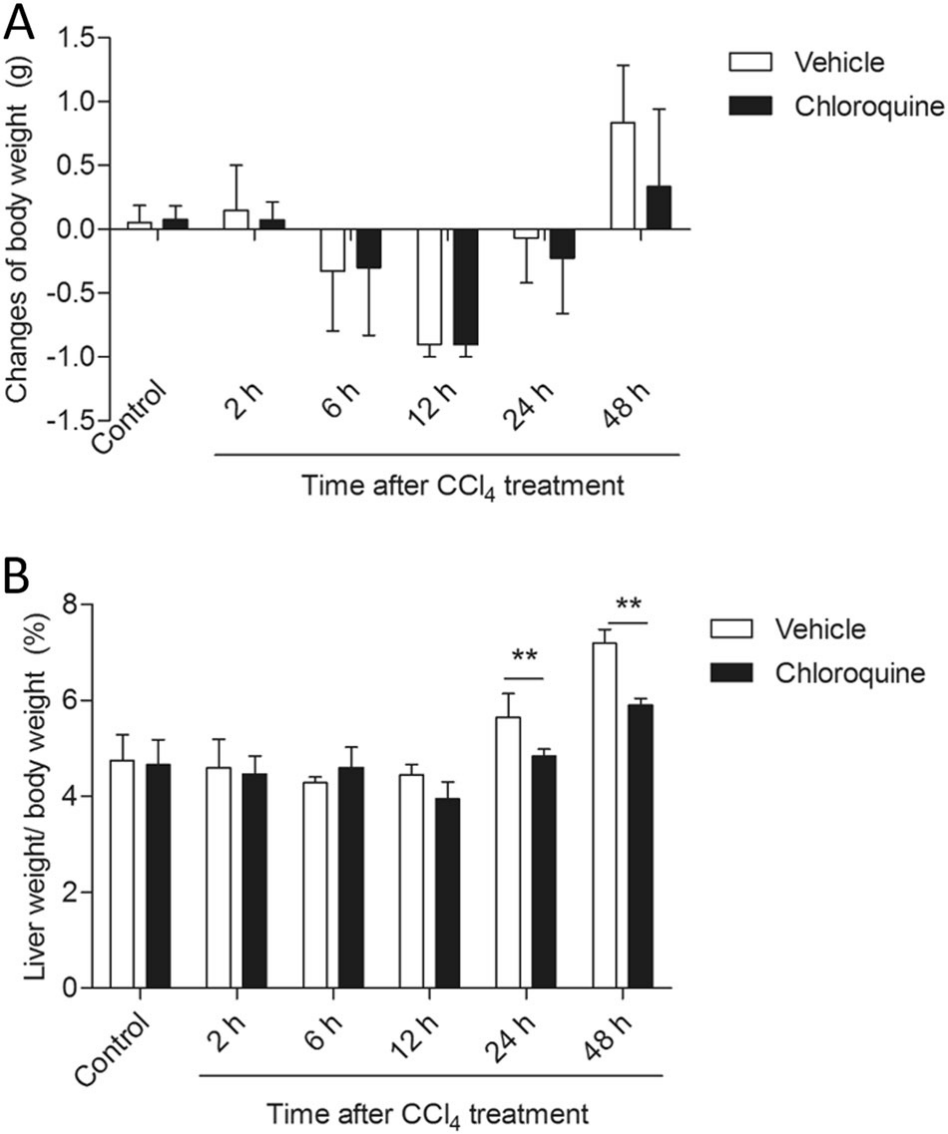
The impact of chloroquine pretreatment on the changes of body weight and liver weight/body weight ratio following CCl4- induced acute liver injury in mice. **a** The changes of body weight. **b** The changes of liver weight/body weight ratio (%). Data are presented as mean ± SD (*n* = 8). **P < 0.01, compared with the CCl_4_ alone group

Histological examination of the livers of mice from CCl_4_ + vehicle group revealed mild liver injury with some cellular necrosis around the blood vessels as early as 2 h. More severe liver injury was evident at 24 h, seen as large areas of extensive cellular necrosis with loss of hepatic architecture and inflammatory cell infiltration around the blood vessels (Fig. 1c). Compared with the vehicle treated control group, the histological scores for the CCl_4_ + vehicle group at 2, 6, 12, 24, and 48 h were all increased to 1.25, 2.25, 2.75, 3.75, and 2.75 (all *P* *<* 0.05 or 0.01), respectively (Fig.1d). CQ pretreatment markedly attenuated CCl_4_-induced liver injury and significantly decreased the histological scores at 2, 6, 12, 24, and 48 h to 0.5, 1.25, 1.75, 2.5, and 1.75, respectively (Fig. 1d). Compared with vehicle-treated control group, there were no abnormal histological changes in the livers of mice from the CQ-only treatment group. The animal’s body weight showed time-dependent changes with the post-time of CCl_4_ treatment (Fig. 2). There were no marked changes between CQ and CQ + CCl_4_ groups at 2, 6, 12, and 24 h. However, at 48 h, there was a significant decrease in the changes of body weight in the CQ + CCl_4_ groups, compared with CCl_4_ group.

**Fig. 3:**
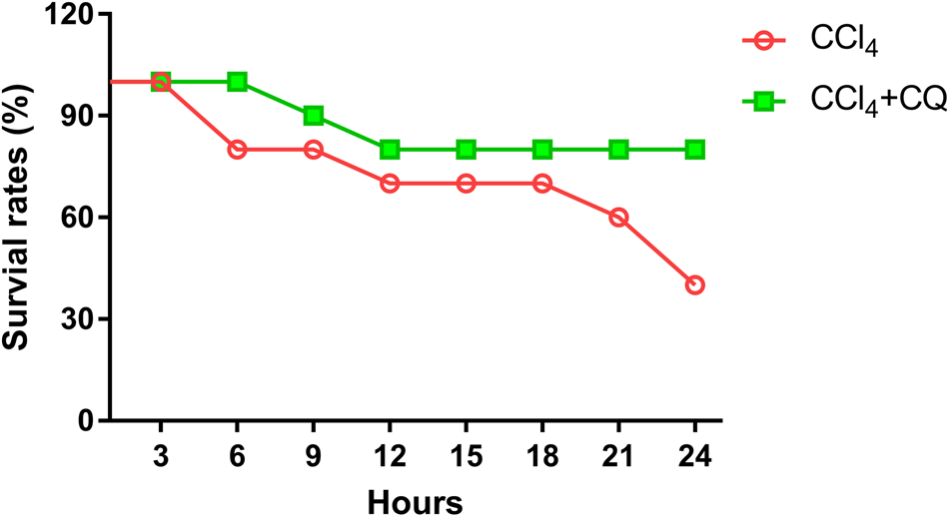
Chloroquine pretreatment attenuates CCl_4_-induced lethal death in mice. In the CCl_4_ group, mice were ip injected with 2.5% CCl_4_ with or withoutpre-treatment of CQ. The death ratios were recorded during 24 h. (*n* = 10 per group)

We further examined the protective role of CQ pretreatment on CCl_4_-induced mortality in mice. The results documented in Fig. 3 showed that the 40% survival rate seen with the CCl_4_ alone treated group improved to 80% at 24 h upon CQ pretreatment in the CQ + CCl_4_ group.

### Chloroquine pre-treatment downregulates CCl_4_-induced hepatic HMGB1 expression and serum levels

**Fig. 4:**
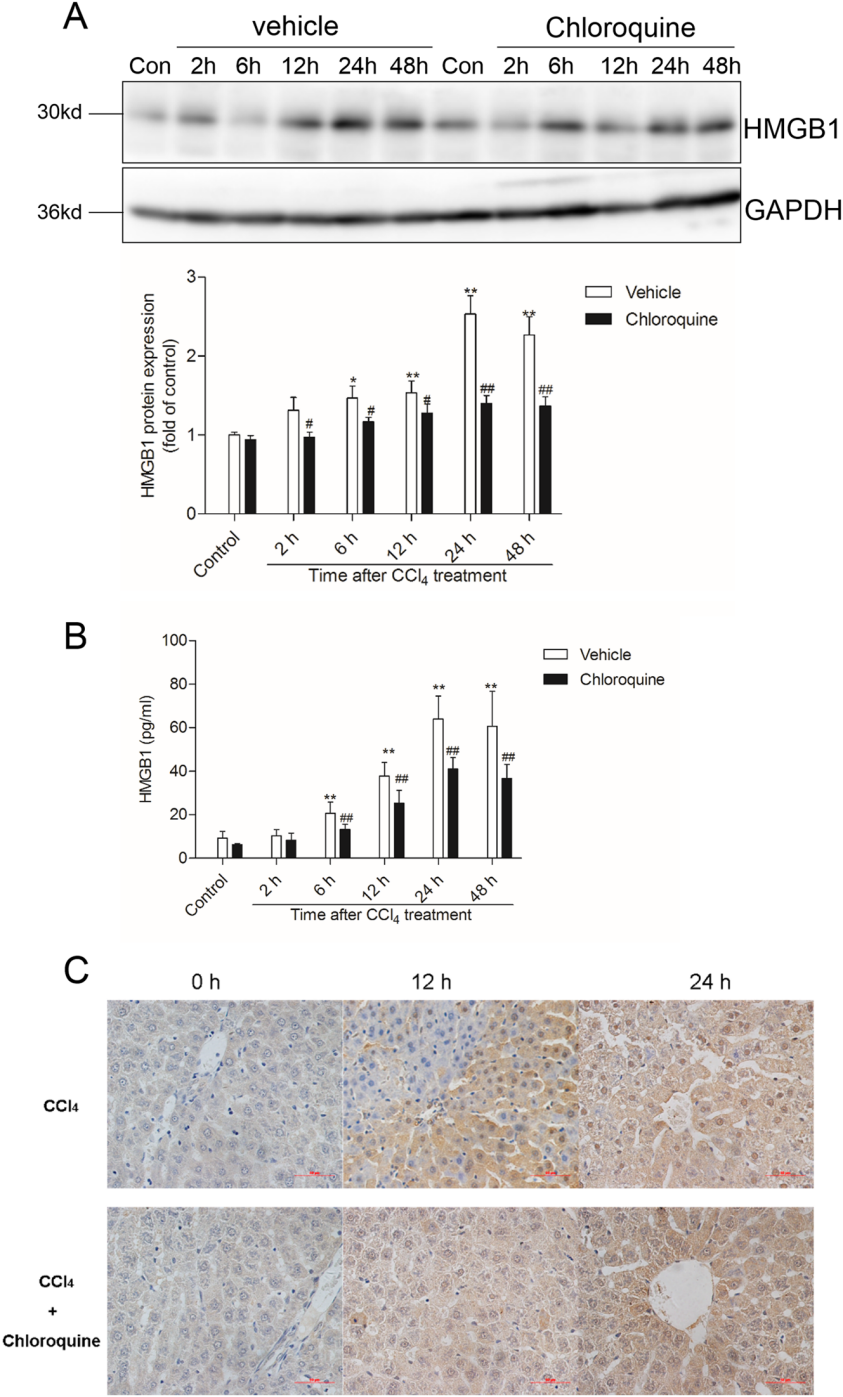
Chloroquine pretreatment downregulates CCl_4_-induced hepatic HMGB1 expression and serum levels. **a** Western blotting analysis of HMGB1 protein expression in the liver tissue of mice from the vehicle and chloroquine (CQ) pretreated CCl_4_ groups (*n* = 4). **b** Serum HMGB1 levels measured by ELISA in mice from the vehicle and CQ pre-treatment groups (*n* = 8) at 24 h. Data are presented as mean ± SD. *P < 0.05 and **P < 0.01, compared with the control group; ^#^*P* < 0.05 and ^##^*P* < 0.01, compared with the CCl_4_ + vehicle group. **c** Representative immunohistochemical results of hepatocytic HMGB1 protein expression at 24 h (*n* = 4). Bar = 50 μm

**Fig. 5:**
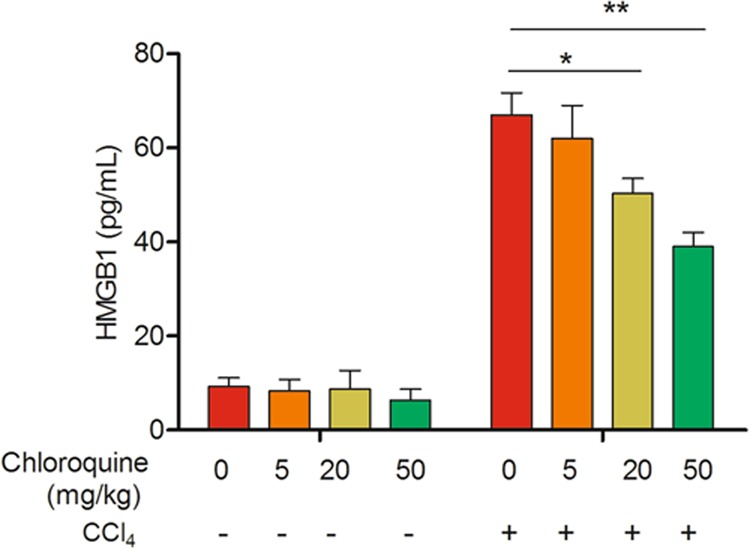
Effect of different dose of chloroquine on the levels of serum HMGB1 in mice exposed with CCl_4_. Serum HMGB1 levels measured by ELISA in mice from the vehicle and CQ pre-treatment groups at 24 h (*n* = 6). Data are presented as mean ± SD. *P < 0.05 and **P < 0.01, compared with the CCl_4_ alone group

In comparison with mice from the vehicle-treated control group, the hepatic tissue expression of HMGB1 and the serum HMGB1 levels were elevated (~2.6- fold at 24 h) in mice from the CCl_4_ + vehicle group (Fig. 4a, b). CQ pre-treatment markedly decreased both CCl_4_-induced hepatic HMGB1 expression and release of HMGB1 into the serum (Fig. 4a, b; Fig. 5). Furthermore, immunohistochemical staining revealed an increase in HMGB1 immunoreactivity and increased HMGB1 translocation from the nucleus to the cytoplasm in liver tissue sections of mice from the CCl_4_ + vehicle group. In comparison, HMGB1 expression in the cytoplasm of hepatocytes significantly decreased in the liver sections of mice from the CCl_4_ + CQ group (Fig. 4c).

### Chloroquine pretreatment inhibits CCl_4_-induced inflammatory responses

**Fig. 6:**
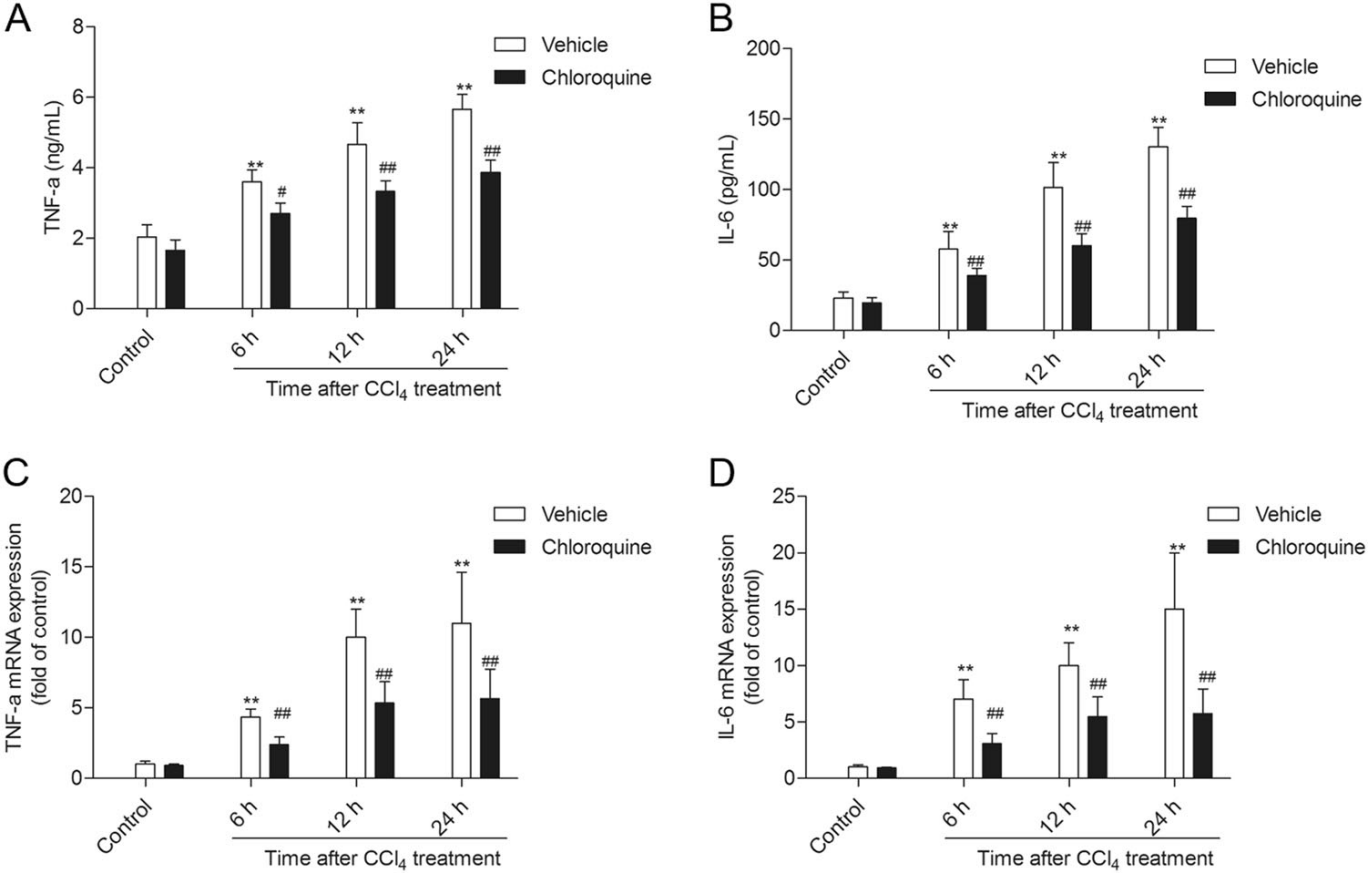
Chloroquine pretreatment attenuates CCl_4_-induced inflammatory responses. **a** Serum levels of TNF-α. **b** Serum levels of IL-6. **c** Hepatic tissue expression levels of TNF-α mRNA. **d** Hepatic tissue expression levels of IL-6 mRNA. Data are presented as mean ± SD (*n* = 8). *P < 0.05 and **P < 0.01, compared with the control group; ^#^*P* < 0.05 and ^##^*P* < 0.01, compared with the CCl_4_ + vehicle group

To examine the impact of CQ pre-treatment on CCl_4_-induced liver inflammatory responses, we measured the serum levels and hepatic mRNA expression of TNF-α and IL-6 (Fig. 6). In comparison with mice from the vehicle-treated control group, mice from the CCl_4_ + vehicle group showed significantly elevated serum levels and hepatic tissue expression of TNF-α and IL-6 mRNAs (Fig. 6). CQ pre-treatment markedly decreased the levels of serum TNF-α (decreased to ~2.5, ~3.4, and ~3.8 ng/mL, at 6, 12, and 24 h, respectively; all *P* *<* 0.05 and 0.01) and IL-6 (decreased to ~39.1, ~60.2, and ~79.7 pg/mL at 6, 12, and 24 h, respectively; all *P* *<* 0.01) (Fig. 6a, b). Similarly, CQ pre-treatment significantly downregulated the CCl_4_-induced hepatic tissue mRNA expression levels of TNF-α and IL-6 (Fig. 6c, d).

### Chloroquine pretreatment modulates the MAPK pathway, NF-KB pathway, autophagy, and apoptosis following CCl_4_-induced acute liver injury in mice

**Fig. 7:**
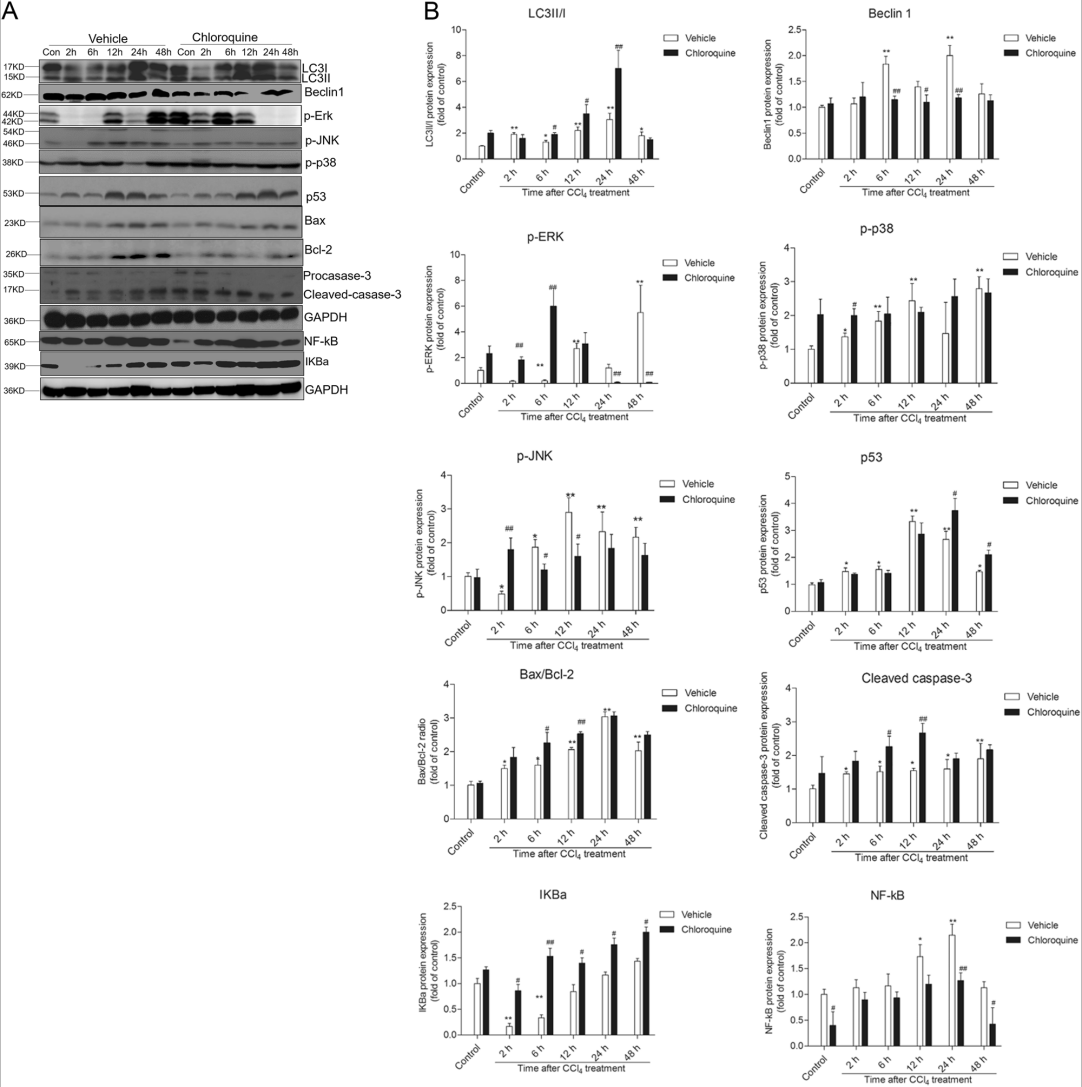
The impact of chloroquine pretreatment on biomarkers of the MAPK family, NF-κB pathway, autophagy, and apoptosis following CCl_4_- induced acute liver injury in mice. **a** Western blot analysis for LC3, Beclin1, phosphorylation (p)-Erk, p- JNK, p-p38, p53, Bax, Bcl-2, NF-κB, IκBa, and cleaved caspase-3 were performed on the liver tissue of mice at 2, 6, 12, 24, and 48 h from the CCl_4_ + vehicle and CCl_4_ + chloroquine (CQ) groups (*n* = 3). **b** The densitometric analysis of the bands was conducted using Image J. Data are shown as mean ± SD (*n* = 3). *P < 0.05 and **P < 0.01, compared with the control group; ^#^*P* < 0.05 and ^##^*P* < 0.01, compared with the CCl_4_ + vehicle group

**Fig. 8:**
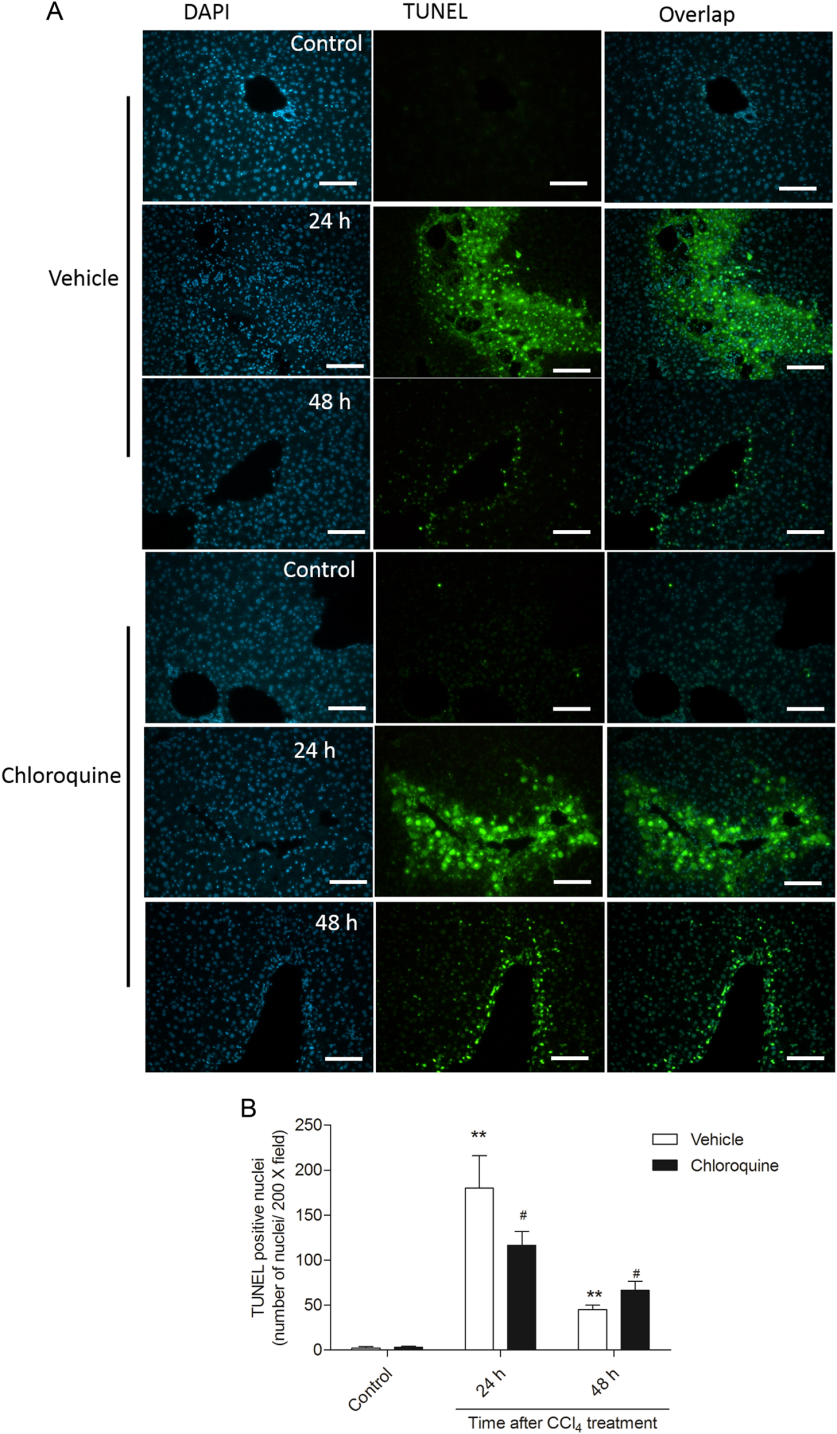
Effect of chloroquine pre-treatment on CCl_4_-induced cell apoptosis in liver tissues. **a** Representative TUNEL-stained sections showing apoptosis in the liver tissue of mice from the vehicle and chloroquine (CQ) pretreated CCl_4_ groups (*n* = 4). Bar = 100 μm. **b** TUNEL-positive cells were counted and statistical analyses presented as mean ± SD. **P < 0.01, compared with the control group; ^#^*P* < 0.05, compared with the CCl_4_ + vehicle group

CCl_4_ treatment significantly increased the expression of the autophagy markers Beclin1 and conversion of LC3II in hepatic tissue of mice at 2, 6, 12, and 24 h (Fig. 7). CQ pre-treatment upregulated the conversion of LC3II and downregulated expression of Beclin1. Notably, expression of Beclin1 and conversion of LC3II recovered at 48 h and no significant differences were evident between CCl_4_ alone and CQ pretreatment groups. For the biomarkers of the MAPK family (p-JNK, p-p38, p-Erk), CCl_4_ treatment increased the hepatic expression of p-JNK at 6, 12, 24, and 48 h relative to the vehicle control. Similarly, the hepatic expression of p-p38 was increased at 2, 6, 12, and 48 h, whereas the expression of p-Erk was increased at 12 and markedly at 48 h. CQ pretreatment produced the opposite effect on p-JNK expression, wherein decreased levels were evident at 6, 12, 24, and 48 h and increased levels were seen at 2 h, relative to the CCl_4_ + vehicle treatment. In the case of p-Erk, CQ pre-treatment increased the expression at 2 and 6 h and markedly decreased levels at 24 and 48 h. The expression of p38 following CQ pretreatment remained relative unchanged compared with the CCl_4_ + vehicle treatment. For the biomarkers of apoptosis, at 2, 6, 12, 24, and 48 h post CCl_4_ treatment, hepatic expression levels of p53, the ratio of Bax/Bcl-2, and cleaved caspase-3 significantly increased. CQ pre-treatment further upregulated the expression of these apoptotic biomarkers. In addition, CCl_4_ treatment upregulated the expression of NF-κB and IκBa at 24 and 48 h. Whereas, CQ pretreatment markedly inhibited the expression of NF-kB and IκBa. TUNEL-stained liver sections confirmed CQ pretreatment increased end stage of apoptosis in the liver tissue of mice, at 48 h relative to the vehicle treated control (Fig. 8). In line with the western blotting data, this effect was less pronounced at 24 h.

### CCl_4_ blocks autophagy flux

**Fig. 9:**
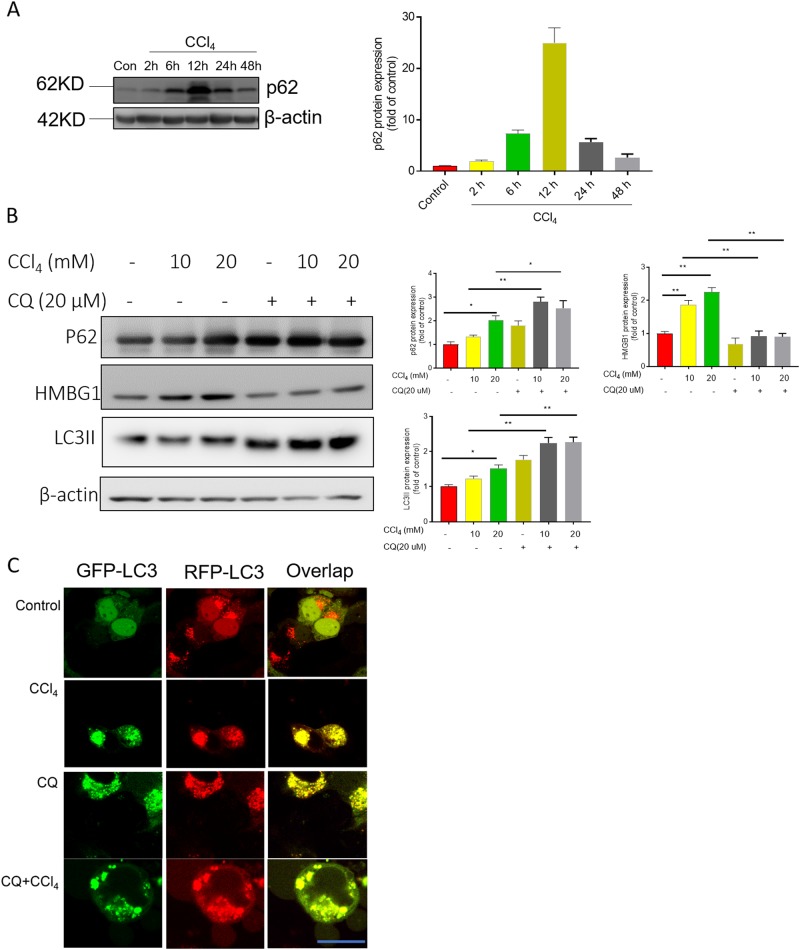
CCl_4_ blocks autophagy flux. **a** p62 protein expression was examined in the liver tissue of mice at 2, 6, 12, 24, and 48 h following CCl_4_ exposure. **b** HMGB1, p62, and LC3II protein expression was examined using western blotting (left panel) and the corresponding analysis (right panel). Data are shown as mean ± SD (*n* = 3). *P < 0.05 and **P < 0.01, compared between different group. **c** CCl_4_ causes accumulation of autophagosomes by inhibiting the later stage of autophagy. Live-cell imaging of HepG2 cells transfected with a tandem mRFP-GFP-LC3 construct for 12 h or treated with 20 μM CQ for 12 h. Bar = 25 μm

In the animal model, CCl_4_ treatment induced dynamic changes in p62 expression (Fig. 9a); At 12 h, p62 protein levels increased ~25 fold, compared with the untreated control. Furthermore, we examined the role of autophagy using a HepG2 cell culture model. As shown in Fig. 8, in HepG2 cells that treated with CCl_4_ (10 or 20 mM), LC3, p62, and HMGB1 protein levels significantly increased. CQ pretreatment further promoted the expression of LC3 and p62 proteins, but significantly inhibited the expression of HMGB1, compared with the CCl_4_ alone treated group (Fig. 9b).

Furthermore, to investigate which stage of the autophagic process is affected, a tandem human red fluorescent protein RFP-GFP-LC3 construct was transfected into the HepG2 cells. The results showed that HepG2 cells treated with CCl_4_ at 20 mM and/or CQ 20 µM for 12 h had mostly yellow/orange puncta indicative of the inhibition of autophagy flux by impairing autophagosome-lysosome fusion (Fig. 9c).

## Discussion

The global burden of liver disease is enormous, with an estimate of just over one million deaths annually^21^. This highlights the need for the discovery of effective “off-the-shelf” hepatoprotective agents. In the current study, we provide demonstrable evidence that pre-treatment with the FDA approved drug CQ at 50 mg/kg for 2 days effectively attenuates CCl_4_-induced acute liver injury in a murine model (Fig. 1).

HMGB1 is an abundant and widely expressed DNA-binding protein that is involved in multiple pathological and physiological processes^5,11,22^. In the course of tissue injury, HMGB1 is secreted from activated immune cells or passively released into the extracellular milieu by dying or injured cells^5,22^. In the present study, we show that the hepatic tissue expression and serum levels of HMGB1 are coincident with the severity of histopathological damage in the livers of mice treated with CCl_4_, and that this effect could be ameliorated by CQ pre-treatment (Figs. 1, 2). Notably, CQ pre-treatment can markedly improve high dose of CCl_4_-induced lethal death in mice (Fig. 3). In line with our findings, previous studies have shown that the systemic injection of a HMGB1-neutralizing antibody could effectively inhibit the inflammatory response and ameliorate CCl_4_- and acetaminophen (APAP)-induced acute liver injury^7,14^. Since, HMGB1 is known to mediate inflammatory signaling related to the aforementioned pathological processess^6–9,15,19,23–25^, it represents an attractive antiinflammatory therapeutic target against acute liver injury^6–9,11,18,25^. In a mouse model of lethal sepsis, it has been demonstrated that CQ inhibits HMGB1-mediated inflammation by blocking NF-κB activation, then improves LPS-induced lethal death in mice^18^. NF-κB activation and nuclear translocation involves IκB kinase (IKK)-mediated phosphorylation IκB-α, which is then released from the NF-κB complex and is subsequently degraded.^18^ The degradation of IκB-α liberates NF-κB, facilitating its nuclear translocation where it activates the expression of its proinflammatory cytokine target genes namely IL-1, IL-2, IL-6, and TNF-α.^11^ In the present study, CQ pre-treatment markedly inhibited the decrease of IκB-α and activation of NF-κB caused by CCl_4_, this in turn cascaded into decreased expression levels of IL-6 and TNF-α in the liver tissue and their circulating serum levels in mice (Figs. 6, 7). Notably, the CQ derivative hydroxyl-chloroquine has been shown to effectively attenuate streptozotocin-induced diabetic renal injury by inhibiting the release of inflammatory cytokines and attenuating apoptotic cell death^26^.

It is well known that the hepatic metabolism of CCl_4_ releases large amounts of reactive oxygen species (ROS), which in turn triggers apoptosis, autophagy, inflammation as well as tissue necrosis^3,14,27,28^. Not surprisingly, the mitochondrial pathway has been shown to play an important role in CCl_4_-induced apoptosis^29^. The mitochondrial pathway is regulated by a balancing act between the pro- and anti-apoptotic Bcl-2 family proteins, including Bax and Bcl-2^30^. Mitochondrial stress leads to the release of CytC into the cytoplasm, which in turn leads to the activation of caspase-9, and −3, which triggers apoptosis^30^. Moreover, p53 can also directly activate Bax, which permeabilizes mitochondria, causing mitochondrial CytC release and caspase activation, which again triggers apoptosis^31^. In the present study, we show that CCl_4_ treatment is followed by a time-dependent increase in the Bax/Bcl-2 ratio, p53 expression, and activation of caspase-3 in hepatic tissue (Fig. 7); and these effects were further enhanced by CQ pre-treatment. The results from TUNEL staining (Fig. 7) at 48 h supported this phenomenon. Interestingly, we detected more TUNEL-positive cells at 24 h in the liver tissue of mice in the CCl_4_ alone treated group, than that in the CCl4 + CQ group (Fig. 8). Notably, DNA damage not only occurs during apoptotic cell death, but can also occur in the latter stages of necrotic cell death.^32,33^ This is where the TUNEL method has a serious drawback, in that it lacks the ability to discriminate apoptotic from necrotic cells given that the latter also have free DNA ends.^32^ Taken together, these results indicate that the observed increase in apoptotic cell death across the total cell population indirectly reflects the protective action of CQ, which inhibits CCl_4_ induced necrotic cell death. The precise cell death dynamics that ensue during these treatments awaits further investigations.

One mechanism whereby the body copes with tissue damage on the cellular level is by employing a fine balance between autophagy and apoptosis^31^. In this elegant mechanism, autophagy acts to prevent or delay apoptotic cell death, and conversely when a cell cannot recover, apoptosis-associated caspase activation shuts off the autophagic process and kills the extensively damaged cell^34^. LC3II and Beclin1 are markers of autophagic flux as they take part in the initiation and closure of the autophagic vesicle, respectively^35^. Our results demonstrate that increased hepatic autophagy follows CCl_4_-induced acute liver injury, which could be effectively attenuated by CQ pretreatment (Fig. 7). CQ appears to block autophagy flux by impairing autophagosome-lysosome fusion (Fig. 9). Notably, the inhibition of autophagy by CQ coincided with enhanced apoptosis at 6 h and 12 h, which was evidenced by the significant increase in the Bax/Bcl-2 ratio and the expression of cleaved caspase-3 (Fig. 7), an indicator for initiation of apoptosis. These data suggest that CQ facilitates the elimination of extensively damaged cells from the liver tissue by promoting the activation of their apoptotic cell death pathways and concomitantly inhibiting autophagy. The knockdown of Beclin1 has been shown to attenuate HMGB1-mediated release of TNF-α and IL-6 in lethal sepsis via inhibiting NF-kB^18^. This would suggest that the downregulation of Beclin1 by CQ may partly contribute to its hepatoprotective effect via the inhibition of HMGB1-mediated inflammatory responses.

The MAPK, such as ERK, JNK, and p38, leads to the transcription of genes regulating cellular response to a plethora of stimuli such as proinflammatory mediators^34,36^. It has been demonstrated that ERK, JNK, and p38 are involved in acute liver injury following treatment with hepatotoxins such as CCl_4_, APAP, or concanavalin A^23,27,37^. In the present study, the expression of p-JNK, p-p38, and p-ERK showed dynamic changes during 48 h after CCl_4_ treatment. In the early phases of CCl_4_-induced acute liver injury (i.e., at 2 h and 6 h), we observed a significant decrease in the expression of p-Erk, which was followed by increased expression levels at 48 h, whereas CQ pretreatment produced the opposite effect (Fig. 7). Taken together, these data suggest that modulating of MAPK activation is also involved in the hepatic protective effects of CQ, which is in line with the proposed role of IL-6/Stat3 signaling for hepatoprotection^38^.

Taken together, our data indicated that CQ protects mice from CCl_4_-induced acute liver injury firstly, through the inhibition of hepatic HMGB1 expression and/or its systemic release, thereby preventing downstream inflammatory events; and secondly by inhibiting autophagy and promoting the apoptotic cells death of non-recoverable cells. Considering CQ is approved for human use, the next step towards the clinical translation of the data would be to perform scientifically based dosing studies of CQ in patients suffering from acute liver disease.

## Materials and methods

### Chemicals and reagents

CCl4 was purchased from Kaixing Chemical Industry Co., Ltd. (Tianjin, China). CQ (purity ≥ 98%) was purchased from Sigma-Aldrich (St. Louis, MO, USA). Sodium dodecyl sulfonate (SDS), aprotinin, leupeptin, pepstatin A, and phenylmethylsulfonyl fluoride (PMSF) were purchased from AMRESCO Inc. (Solon, OH, USA). Dulbecco’s modified Eagle’s medium (DMEM) and fetal bovine serum (FBS) were from Life Technologies Corporation (Grand Island, NY, USA). All other chemicals were of the highest analytical grade available.

### Animals

C57BL/6 mice (male, 6–8 weeks, 18–22 g) were purchased from Vital River Animal Technology Co., Ltd. (Beijing, China). Mice were housed in a room maintained at a temperature of 23 ± 2 °C and relative humidity of 50 ± 10% with a 12 h light-dark cycle. Mice were acclimatized for 1 week prior to use and had free access to food and water during the entire experiment. All animal experiments were approved by the Institutional Animal Care and Use Committee at the China Agricultural University.

### Cell culture

The human hepatoma HepG2 cell line (American Type Culture Collection (ATCC), HB-8065) was purchased from Cell Bank of Type Culture Collection of Chinese Academy of Sciences (Shanghai, China) and cultured in MEM supplemented with 10% (v/v) heat-inactivated FCS, 110 mg/L sodium pyruvate, 100 units/mL penicillin, and 100 μg/mL streptomycin. Cells were maintained in a humidified atmosphere of 95% air and 5% CO_2_ at 37 °C.

### Experimental design

To investigate the dose-dependent effect of CQ on CCl4-induced release of HMGB1, 48 mice were randomly divided into the following groups: control, CQ5, CQ20, CQ50, CCl_4_, CCl_4_ + CQ5, CCl_4_ + CQ20, and CCl_4_ + CQ50 (*n* = 6 in each group). In the CCl_4_ group, mice were intraperitoneally (i.p) injected with 0.3% CCl_4_ (10 mL/kg, dissolved in olive oil). In the CCl_4_ + CQ5, CCl_4_ + CQ20 and CCl_4_ + CQ50 groups, mice were at 5, 20, and 50 mg/kg at 2, 24, and 48 h prior to CCl_4_ administration. The mice in the control and CQ groups were administrated with an equal volume of vehicle or CQ (5, 20, and 50 mg/kg). After 24 h, mice were anesthetized and blood was collected; the animals were then killed and their livers were harvested. Blood samples were centrifuged at 3000 × *g* for 10 min, and the serum was collected for examining the levels of serum HMGB1.

Then, 96 mice were randomly divided into the following groups: control, CQ, CCl_4_, and CCl_4_ + CQ. In the CCl_4_ group, mice were i.p. injected with 0.3% CCl_4_ (10 mL/kg, dissolved in olive oil). In the CCl_4_ + CQ group, mice were i.p. injected with CQ at 50 mg/kg at 2, 24, and 48 h prior to CCl_4_ administration. The mice in the control and CQ groups were administrated with an equal volume of vehicle or CQ (50 mg/kg). At 2, 6, 12, 24, and 48 h after CCl_4_ administration, mice (*n* = 8 per time point) were anesthetized and blood was collected. Livers were divided into sections and fixed in 10% neutral buffered formalin at room temperature or snap frozen in liquid nitrogen and stored at −80 °C.

To investigate the effect of CQ on CCl_4_-induced lethality, 20 mice were used and divided into the CCl_4_ treatment only group and CCl4 + CQ group. The CQ treatment is same as that mentioned above. Mice were i.p. injected with 2.5% CCl_4_ (10 mL/kg, dissolved in olive oil). Observation of lethality were subsequently performed performed over 24 h.

### Serum transaminase (ALT), aspartate transaminase (AST) assays

The levels of serum ALT and AST were examined by using an Automated Chemical Analyzer (Hitachi 7080, Hitachi High-Technologies Corporation) with the standard diagnostic kits (Shanghai Kehua Bio-engineering Co., Ltd., Shanghai, China).

### Histological examination of liver damage

Livers were randomly selected from four mice and fixed in 10% neutral buffered formalin for 48 h. The samples were dewaxed in xylene and rehydrated in a series of graded alcohols and then embedded in paraffin. The samples were sectioned at 4 μm and stained with hematoxylin–eosin (H&E) for light microscopic examination. To evaluate the degree of necrosis after acute liver injury, an injury grading score (Grade 0–4) based on severity of necrotic lesions in the liver parenchyma were carried out as previously reported^39^. Each sample was independently scored by 3 pathologists who will be blind to the treatment and untreated control groups. The scoring system was as follows: Grade 0, no pathological change; Grade 1, presence of degenerated hepatocytes with only rare foci of necrosis; Grade 2, small area of mild centrilobular necrosis around the central vein; Grade 3, area of mild centrilobular necrosis severer than Grade 2; and Grade 4, centrilobular necrosis severer than Grade 3.

### Cytokine measurement

The serum levels of TNF-α, IL-6, and HMGB1 were measured using enzyme-linked immunosorbent assay (ELISA) kits according to the manufacturer’s instructions (R&D Systems, Minneapolis, MN, USA).

### Immunohistochemical staining

The paraffin-embedded sections of liver tissues from the histopathological evaluations (above) were employed for immunohistochemical experiments. The embedded liver sections were deparaffinized and dehydrated in graded alcohol, then treated with 3% H_2_O_2_ for 15 min, followed by microwave antigen retrieval for another 15 min in citrate buffer. Nonspecific antigens were blocked with 5% goat serum for 30 min. For HMGB1 staining, the specimens were incubated with a rabbit anti-mouse HMGB1 polyclonal antibody (1:200; ProteinTech Group, Inc., Chicago, IL, USA) at 4 °C overnight, followed by a 30 min incubation with a horseradish peroxidase (HRP)-conjugated goat anti-rabbit secondary antibody (Zhongshan Golden Bridge Biotechnology CO. LTD, China). Subsequently, the samples were stained with diaminobenzidine chromogen solution (Zhongshan Cambridge Reagent Company, Beijing, China), followed by hematoxylin, and mounted in xylene-based mountant.

### TUNEL assay

Cell apoptosis in the liver tissue was detected using a terminal deoxynucleotidyl transferase-mediated dUTP nick-end labeling (TUNEL) assay kit, according to the manufacturer’s protocol (Vazyme Biotech Co., Ltd, Nanjing, China). After TUNNEL labeling, the liver sections were counterstained with 4′-6-diamidino-2-phenylindole^40^ to label the nuclei. Images were observed under a fluorescence microscope (Leica Microsystems, Wetzlar, Germany).

### RFP-GFP-LC3 plasmid transfection

HepG2 cells were transiently transfected with the RFP-GFP-LC3 vector, kindly provided by Dr. Shen Zhang (UT southwestern medical Centre, Dallas, TX, USA) using X-treme GENE HP DNA transfection reagents (Roche, Switzerland). After 48 h, cells were treated with CCl_4_ at 20 mM or CQ at 20 μM for 12 h, and the images were captured with a Zeiss Observer.Z1 microscope by using the Slidebook 4.2.0.11 computer program.

### Western blotting

The liver tissue of three mice from each group were lysed using ice-cold lysis buffer (100 mM Tris-HCl, 2% [w/v] SDS, 10% [v/v] glycerol, pH 7.4); protease inhibitor cocktail (1 mM PMSF, 1 μg/mL aprotinin, 1 μg/mL leupeptin, and 1 μg/mL pepstatin A) was added to the lysis buffer before treatment. The samples were ultrasonicated (5 s ultrasonication and 6 s pause in each cycle for 5x, power 30 W) using an Ultrasonic Processor (Branson, MO, USA). The tissue lysates were centrifuged at 14,000 × *g* for 15 min at 4 °C, and the supernatants were collected. The protein concentration was measured using the BCA protein assay kit. Equal amounts of protein from each sample were resolved by SDS-PAGE and transferred to nitrocellulose membranes (Bio-Rad, Hemel Hempstead, UK). To investigate the role of autophagy, HepG2 cells were treated with CQ at 20 μM at 2 h prior to CCl_4_ treatment (10 or 20 mM); after 12 h, the cells were collected and the following protein levels were examined. The following primary antibodies were employed: primary rabbit antibodies against microtubule-associated protein 1 light chain 3 (LC3) (1:1000), Bax (1:1000), NF-κB (1:1000), IκBa (1:1000), Bcl-2 (1:1000) (ProteinTech Group, Inc., Chicago, IL, USA), phosphor (p)-extracellular signal-regulated kinase (ERK) (Thr202/Tyr204) (1:1,000), p-p38 (Thr180/Tyr182), MAPK (1:1000), Beclin1 (1:1000), p-c-Jun N-terminal kinase (JNK) (Thr183/Tyr185) (1:1000) (Cell Signaling Technology, Beverly, MA, USA), caspase-3, p62/SQSTM1 (1:5000), mouse monoclonal antibody against p53 (1:1000), and β-actin (1:1000), glyceraldehyde-3-phosphate dehydrogenase (GAPDH) (1:1000) (Santa Cruz Biotechnology, CA, USA). Peroxidase-conjugated goat anti-rabbit or anti-mouse IgG (1:5000) (Santa Cruz Biotechnology, CA, USA) were employed as the secondary antibodies. The specific protein bands were visualized using the enhanced western luminescent detection kit (Vigorous Biotechnology, Beijing, China). The results were quantified by densitometry using Image J software, and the densitometry results were normalized relative to the GAPDH or β-actin bands.

### RNA extraction and real-time quantitative PCR

Total RNA was isolated using the TRIzol extraction kits according to the manufacturer’s instructions (Invitrogen Inc., Carlsbad, CA, USA). The quality of RNA was verified by evaluating the absorbance at 260 nm and 280 nm. The production of cDNA was obtained from total RNA by using prime scriptTM RT reagent kit (TaKaRa). RT-PCR was performed with SYBR Green qPCR Kit (TaKaRa). The PCR conditions and primers used were as follows: TNF-α forward: 5′-GGC AGG TCT ACT TTG GAG TCA TTG C-3′, TNF-α reverse: 5′-ACA TTC GAG GCT CCA GTG AAT TCG G-3′, IL-6 forward: 5′-TGG AGT CAC AGA AGG AGT GGC TAA G-3′, IL-6 reverse: 5′-TCT GAC CAC AGT GAG GAA TGT CCA C-3′, GAPDH forward, 5′-ACA GTC CAT GCC ATC ACT GCC-3′, GAPDH reverse: 5′-GCC TGC TTC ACC ACC TTC TTG-3′. PCR reactions were run under the following conditions: initial activation of Taq DNA polymerase at 95 °C for 5 min, 40 cycles of 30 s at 95 °C for denaturing, 30 s at 60 °C for annealing, and 30 s at 72 °C for elongation. RT-PCR test was analyzed by ABI QuantStudio™7 detection system (Applied Biosystem, USA). All reactions were conducted in triplicate. GAPDH was used as an internal control, and fold change in gene expression was calculated using the threshold cycle method (2^−ΔΔ*CT*^)^41^.

### Statistical analyses

All Data are presented as mean ± SEM. The statistical analyses were performed using SPSS V16.0 (SPSS Inc., Chicago, IL, USA) and the differences between groups were compared with one-way ANOVA followed by Dunnett’s multiple comparison procedure. A *P*-value < 0.05 were considered as statistically significant.
